# A prospective study on an innovative online forum for peer reviewing of surgical science

**DOI:** 10.1371/journal.pone.0179031

**Published:** 2017-06-29

**Authors:** Martin Almquist, Regula S. von Allmen, Dan Carradice, Steven J. Oosterling, Kirsty McFarlane, Bas Wijnhoven

**Affiliations:** 1 Dept of Surgery, Skåne University Hospital, Lund, Sweden; Institution of Clinical Sciences, Lund University, Lund, Sweden; 2 Clinic for Vascular Surgery, Kantonsspital St Gallen, Switzerland; 3 Academic Vascular Surgical Unit, Hull York Medical School, Hull, United Kingdom; 4 Dept of Surgery, Spaarne Gasthuis Hospital, Haarlem, The Netherlands; 5 Associate Editor, BJS, John Wiley and Sons, Oxford, United Kingdom; 6 Department of Surgery, Erasmus MC–University Medical Center Rotterdam, The Netherlands; Johannes Gutenberg Universitat Mainz, GERMANY

## Abstract

**Background:**

Peer review is important to the scientific process. However, the present system has been criticised and accused of bias, lack of transparency, failure to detect significant breakthrough and error. At the British Journal of Surgery (BJS), after surveying authors’ and reviewers’ opinions on peer review, we piloted an open online forum with the aim of improving the peer review process.

**Methods:**

In December 2014, a web-based survey assessing attitudes towards open online review was sent to reviewers with a BJS account in Scholar One. From April to June 2015, authors were invited to allow their manuscripts to undergo online peer review in addition to the standard peer review process. The quality of each review was evaluated by editors and editorial assistants using a validated instrument based on a Likert scale.

**Results:**

The survey was sent to 6635 reviewers. In all, 1454 (21.9%) responded. Support for online peer review was strong, with only 10% stating that they would not subject their manuscripts to online peer review. The most prevalent concern was about intellectual property, being highlighted in 118 of 284 comments (41.5%). Out of 265 eligible manuscripts, 110 were included in the online peer review trial. Around 7000 potential reviewers were invited to review each manuscript. In all, 44 of 110 manuscripts (40%) received 100 reviews from 59 reviewers, alongside 115 conventional reviews. The quality of the open forum reviews was lower than for conventional reviews (2.13 (± 0.75) versus 2.84 (± 0.71), P<0.001).

**Conclusion:**

Open online peer review is feasible in this setting, but it attracts few reviews, of lower quality than conventional peer reviews.

## Introduction

Formal peer review has been used by many scientific journals since the 18th century. It is regarded as a trusted form of scientific evaluation, which ideally guarantees and improves scientific reporting, thus serving as a quality-control system. To corroborate this there is empirical evidence that conventional peer review improves the quality of papers [1, 2] and predicts future impact [3–5]. There is, however, also evidence of the failings of conventional peer review, including bias [5–13], failure to identify error and fraud [14–17] and suppression of innovation [18]. In a survey among 4000 international authors, two-thirds felt that conventional peer review could be improved [19].

A change in the traditional peer review process seems inevitable and has already been advocated by others [20]. Peer review should focus on improving the quality of a paper as well as improving transparency and objectivity. Although this is a significant challenge, the time has come to explore new systems to establish whether the aims of peer review may be better served and whether modernisation of peer review may have any additional benefits to medical publishing.

Thus, the aim of this study was to investigate attitudes towards modernisation, the advantages and disadvantages of an innovative online peer review system in comparison with traditional peer review and to obtain the views of those involved in this new process.

## Methods

The British Journal of Surgery (BJS) is a monthly, peer-reviewed surgical journal, with a current five-year impact factor of 5.596 (2015). BJS publishes original work in a range of surgical specialties. The journal receives approximately 1800–2000 manuscripts annually. The acceptance rate in 2015 was 12%.

### BJS standard peer review process

In the BJS standard peer review process, the co-Editor-in-Chief assesses the manuscript for suitability and a small percentage of manuscripts are rejected based on article type. All manuscripts are then allocated to a handling editor, who either selects two to three relevant referees for peer review or consults with at least one other editor before rejecting the manuscript without peer review [21]. The process is single-blinded: the author’s identity is known to the reviewer but the reviewer’s name is not disclosed to the author. Referees are asked to submit the review within two weeks, alongside a recommendation that the paper be accepted, revised or rejected. Referees use an online evaluation form (S1 Fig). The handling editor scores the reports of the referees as cursory (1 point), inadequate (2 points), adequate (3 points), good (4 points) or outstanding (5 points). If the first two referee reports are in agreement, the handling editor makes a decision on the basis of these two reports. If the opinions of two referees differ significantly, the handling editor will invite additional referees. The decision is communicated as soon as possible to the author [21]. The process is managed using the ScholarOne^TM^ platform (Thomson Reuters). The mean time from submission to decision in 2014 was 25 days.

### Innovative online peer review process

The intention was to develop an innovative peer review system that took full advantage of modern technology to improve the peer review process and draw reviewers and readers into meaningful online scientific debate. The key elements would be: instant electronic real-time communication; an electronic forum; and social media integration, promoting awareness, escalating engagement and propagating participation. The aims of such a system are to: improve the quality of peer review and subsequent post-review manuscripts; draw on a wider range of potential peer reviewers; promote transparency; provide an inside view on critical appraisal, research and peer review to junior surgeons and researchers; and increase readership and impact of the journal and its research.

### Study design

The present study was divided into three parts: a pre-trial questionnaire, a prospective study on an innovative online forum for peer reviewing and a post-trial questionnaire.

### Pre-trial questionnaire

The pre-trial questionnaire aimed to investigate the opinion of authors and reviewers towards an online forum for peer review, and to introduce authors and reviewers to the idea of using an online forum for peer review. The questionnaire was conducted in December 2014, using SurveyMonkey [22]. Information sought included respondents’ gender, age, native language and previous experience with scientific writing and reviewing. The willingness to use an innovative online forum for peer reviewing, both as potential author and reviewer, and the reason for not wanting to use such a forum were also evaluated.

The questionnaire (S2 Fig) was circulated by email to reviewers with an accredited account in the BJS ScholarOne system. Completion was anonymous and it was possible to leave items blank. The questionnaire was distributed once, with no follow-up correspondence.

### Prospective study on an online forum for peer review

All manuscripts involving original work (i.e. observational studies, randomised controlled studies and systematic reviews) submitted to BJS between 1^st^ April and 31^st^ May 2015 were considered for possible inclusion. Upon submission the author was informed about the study and their consent for participation was sought. It was made clear to authors that participation was voluntary and would not affect the outcome of their submission. Manuscripts from consenting authors simultaneously went through standard peer review and innovative peer review using an online forum. Manuscripts were posted online, and an invitation to review these online manuscripts was sent every 14 days by email to approximately 7000 referees with an accredited account in BJS ScholarOne. The manuscript was kept online for 3 weeks and was only accessible through the link supplied in the email.

Referees reviewing on the online forum were asked to report using the same standard referee sheets as in the standard peer review process (S1 Fig). Completed reports were automatically saved in Google Docs and this was checked every day to see if any new reports had been submitted. At that point, the “comments to authors” section from the scoresheet was uploaded to the manuscript online and the comments were also sent to the authors. The authors were able to view the open reviews while their manuscript was still undergoing traditional review, and could comment on the reviews if they wished. Authors were not blinded to the identity of the reviewers submitting reviews via the online system; it was not possible to submit reviews anonymously.

According to the pre-specified study protocol, the handling editor decided on the manuscript based on the standard peer review only, without viewing the reports generated in the online forum. After the editorial decision, the online peer reviews were sent to the handling editor, who scored them according to the standard BJS policy rating system, based on a 5-point Likert scale, from cursory (1 point) to outstanding (5 points), as defined above.

**Fig 1:**
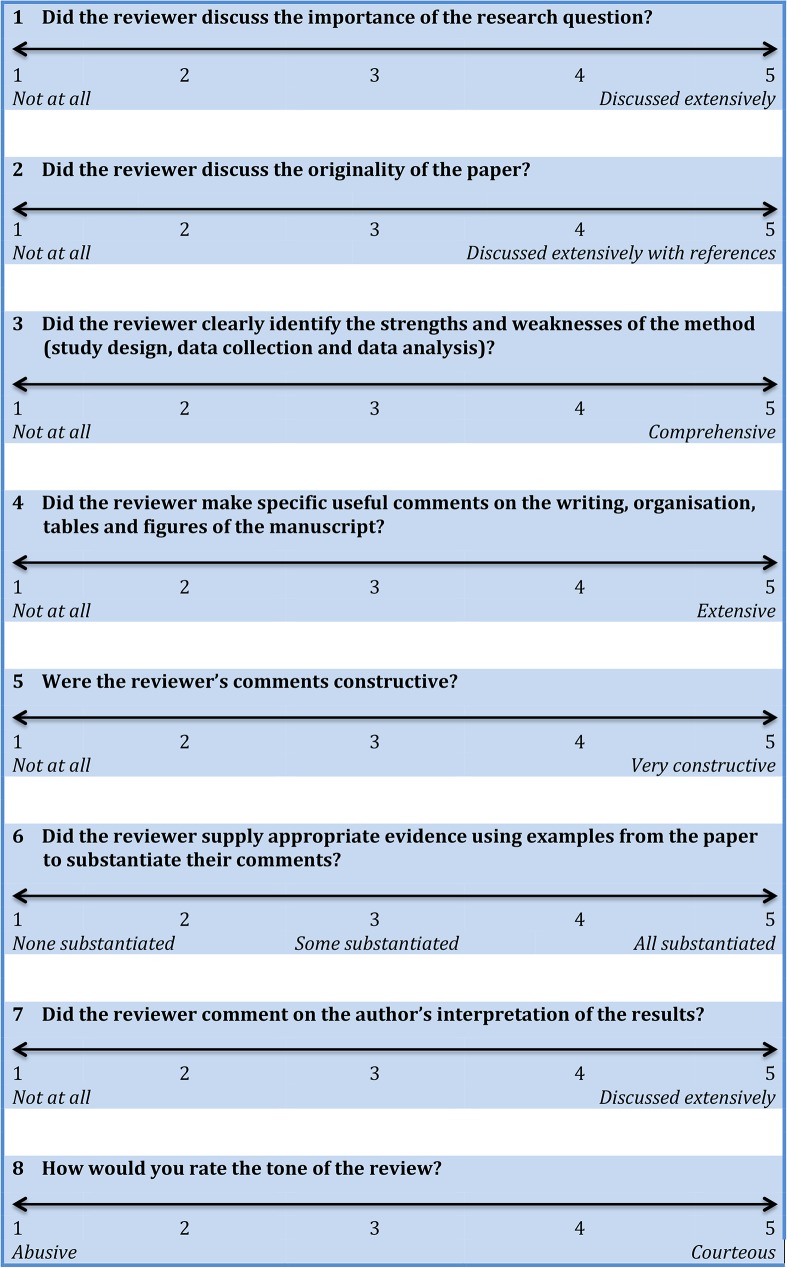
Review quality instrument by van Rooyen et al. [23].

In parallel, two editorial assistants (MA, RvA) assessed all reviews, both from the conventional peer review process and from the innovative online forum, using a previously validated instrument by van Rooyen *et al*. (Fig 1) [23]. This instrument consists of seven questions relating to: importance of the research question; originality; method; presentation; constructiveness of comments; substantiation of comments; and interpretation of results. Each of the seven items were scored in a 5-point Likert scale from 1 (= poor) to 5 (= excellent). An additional item assessed the overall tone of the review, ranging from abusive to courteous. A total score was based on the mean of the eight items’ scores.

In order to secure the authors’ copyright during the study, the line “Copyright 2015 The Authors. All rights reserved.” was added at the top of each manuscript. If a manuscript was accepted, standard BJS policy was applied, and the corresponding author received an email prompting him to log in to Author Services, where, via the Wiley Author Licensing Service (WALS), the license agreement had to be completed on behalf of all authors on the paper.

The primary outcome of the present study was the quality of referee reports based on the validated review quality instrument (Fig 1). For the primary outcome, only those manuscripts that received reviews from the open online forum were included. Secondary outcomes were participation rate, i.e. number of reviews and/or author comments per paper in the open online forum, and quality of open and standard referee reports, based on the BJS scoring system for peer review quality.

### Post-trial questionnaire

The aim of the post-trial questionnaire was to assess the overall satisfaction of authors with the online review process. A web-based questionnaire [22] was sent to participating authors upon study completion on 2^nd^ September 2015. Specific survey questions are shown in S3 Fig.

### Statistical analysis

Responses to the pre-trial questionnaire were summarised using descriptive statistics. Factors influencing the likelihood of a respondent’s participation in online peer review were analysed using single and multiple logistic regression, testing associations between characteristics of respondents and responding yes to “would you consider participating in open online peer review”, yielding odds ratios (OR) with 95% confidence intervals (CI). In the multiple models, all items in the questionnaire were included (S2 Fig) as categorical co-variates, except for the free-text item and the item “would you comment on online papers”. Responses with missing data were not included in the regression analyses.

A thematic analysis was performed for the only free-text answer and was narratively summarised.

Review quality scores from editors (using a 1–5 point scale) and from assistant editors (using the 8-item validated checklist) were summarised and compared between standard and online review using means and Student t-tests. Agreement between assistant editors’ scoring was measured using kappa statistics. A *P* value of <0.05 was considered statistically significant.

### Ethics

Ethical approval was not sought, as patients were not directly involved. It was assumed that consent was given when participants actively chose to participate in the survey. Authors submitting their work received information about the study at submission, and it was made clear to them that participation was voluntary and would not affect the outcome of their manuscript. Authors consented to participating by ticking a box at the submission site.

## Results

### Pre-trial questionnaire

The pre-trial questionnaire was sent to 6635 listed reviewers. There were 1454 individual responses, corresponding to a response rate of 21.9%. Not every item in the questionnaire was answered by all respondents, but missing responses were few: no more than 2.5% for any item.

Some 87% of respondents were male, 94% of the respondents already acted as a reviewer and 78% had published and/or submitted more than 20 papers (Table 1). The majority of the respondents supported an experimental online peer review study as an author, with only 10% indicating that they would not participate. Support for participation as a reviewer was less positive, with 18% stating that they would not review online and 40% being unsure.

**Table 1:** Characteristics of respondents to the pre-trial questionnaire according to willingness to participate in open peer review. Numbers (column per cent).

		All (n = 1454)	Would participate (n = 973)	OR (95% CI)
**Sex**	Male	1261 (87.5)	870 (89.9)	1.99 (1.43–2.78)
	Female	180 (12.5)	98 (10.1)	1.00
**Age**	<30	22 (1.5)	14 (1.4)	0.89 (0.39–2.39)
	30–40	232 (16.0)	166 (17.1)	1.00
	40–50	421 (29.0)	265 (27.4)	0.67 (0.47–0.96)
	50–60	462 (31.9)	311 (32.1)	0.83 (0.57–1.20)
	>60	312 (21.5)	21 (21.9)	0.83 (0.55–1.24)
**First language**	English	602 (41.8)	425 (44.0)	1.33 (1.06–1.68)
	Other	839 (58.2)	542 (56.0)	1.00
**No. of papers previously submitted/ published**	None	7 (0.5)	3 (0.3)	0.57 (0.10–3.12
	1–5	80 (5.5)	51 (5.2)	1.00
	5–20	225 (15.6)	160 (16.5)	1.23 (0.69–2.17)
	>20	1133 (78.4)	757 (78.0)	0.99 (0.58–1.69)
**Previous submission to BJS**	Yes	1118 (77.6)	771 (79.4)	1.32 (1.01–1.72)
	No	322 (22.4)	200 (20.6)	1.00
**Number of annual reviews**	None	80 (5.6)	54 (5.6)	1.02 (0.58–1.80)
	<10	540 (37.5)	365 (37.6)	0.96 (0.72–1.28)
	10–20	438 (30.4)	307 (31.6)	1.00
	>20	381 (26.5)	244 (25.2)	0.78 (0.58–1.05)
**Would you submit to open peer review**	Yes	973 (67.6)	-	-
	No	145 (10.1)	-	-
	Unsure	321 (22.3)	-	-
**Would you comment on on-line papers**	Yes	595 (42.1)	505 (52.8)	-
	No	252 (17.8)	100 (10.5)	-
	Unsure	565 (40.0)	351 (36.7)	-

Numbers do not always add up since it was possible to leave item blank

Multiple logistic regression with odds ratios and 95% confidence interval for answering yes to “would you submit to open peer review”, adjusted for sex, age, first language, nr of previous published and/or submitted papers, previous submission to BJS, and nr of annual reviews

Free-text answers regarding thoughts and concerns about the online peer review process are summarised in Table 2. The most prevalent concern was about intellectual property issues, being highlighted in 118 of 284 comments (41.5%). Still, the majority of respondents indicated that they were willing to participate in an open peer review trial.

**Table 2:** Collated concerns and free text responses from survey respondents.

Categories of concerns and comments	N = 284*
**Intellectual property**	
** Risk of ideas or data theft, n (%)**	118 (41.5)
** Uncertainty about acceptance by another journal after rejection by BJS, n (%)**	15 (5)
** Conflict of interest difficulties, n (%)**	5 (2)
**Quality of reviews**	
** Low quality; deliberate “bad talking”, or unduly favourable reviews for associates, n (%)**	61 (21.5)
**Procedural concerns**	
** Prolonged time to decision: Competitors may gain an advantage, n (%)**	15 (5)
** Increased workload for authors and reviewers, n (%)**	31 (11)
**Concerns about anonymity of referees, n (%)**	5 (2)
**Present review process should not be revised, n (%)**	12 (4)
**Comments regarding the novel peer review study**	
** Concerns and recommendations regarding study methodology, n (%)**	6 (2)
**Uncertainties about the value and proceeding of the novel review study, n (%)**	22 (8)
**Supporting comments, n (%)**	4 (1.5)
**Other**	
** Unrelated to the proposed study, n (%)**	7 (2.5)

* Multiple concerns per response were raised, therefore total number of concerns do not add up to total number of responses

In multiple regression analysis, male gender, English as first language and previous submission to BJS were all associated with willingness to participate in online peer review (Table 1).

### Prospective study

**Fig 2:**
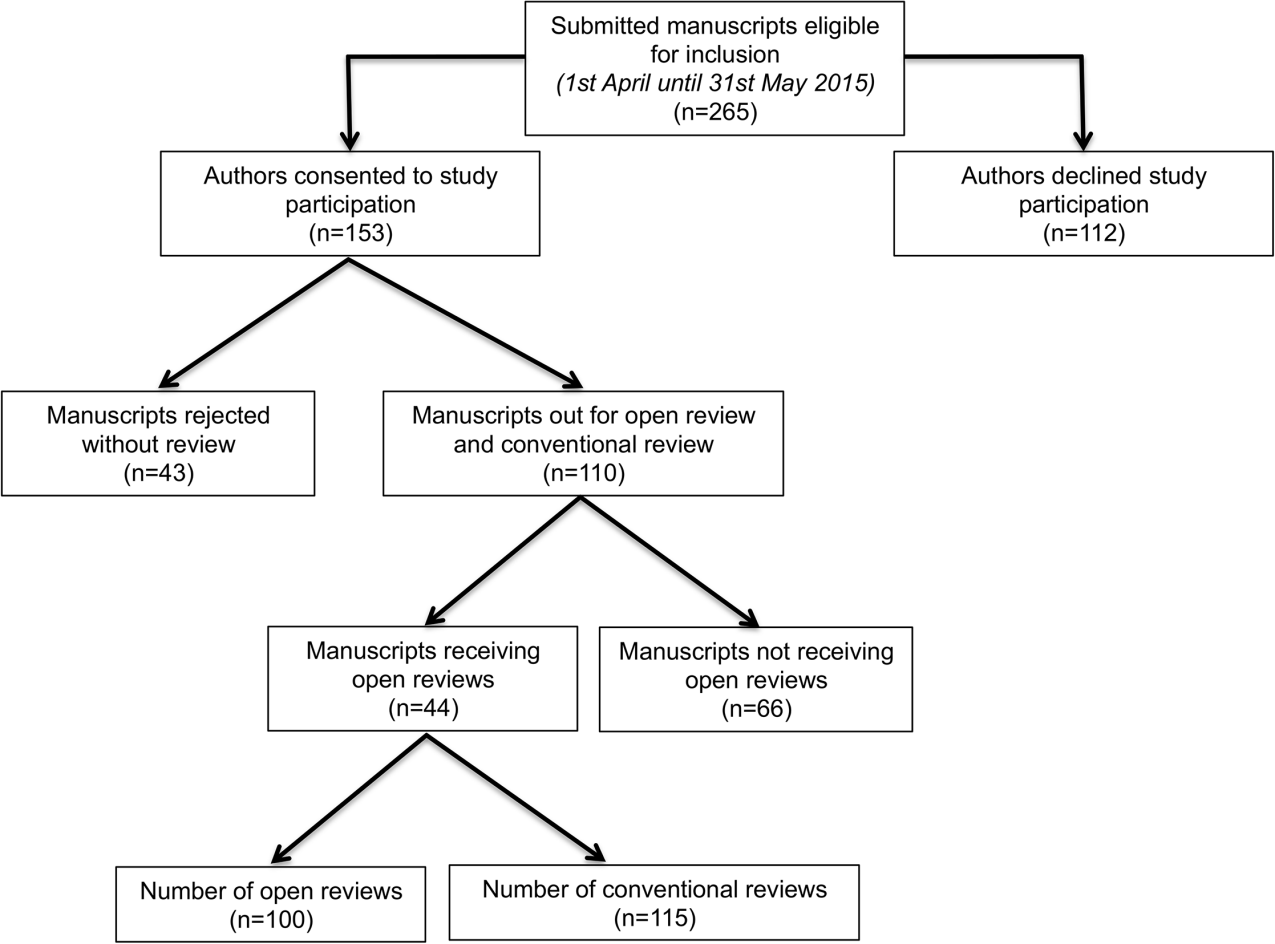
Flowchart of manuscripts included in study.

Between 1^st^ April and 31^st^ May 2015 a total of 295 manuscripts, including Leading Articles, Snapshots in Surgery, and Original Articles were submitted to the BJS; of these, 265 were eligible for the study (Fig 2). Some 110 manuscripts were included in the online peer review trial. Of these 110, only 44 (40%) received at least one review. The total number of reviews for these 44 manuscripts was 100, and the total number of conventional reviews for the same 44 manuscripts was 115.

**Fig 3:**
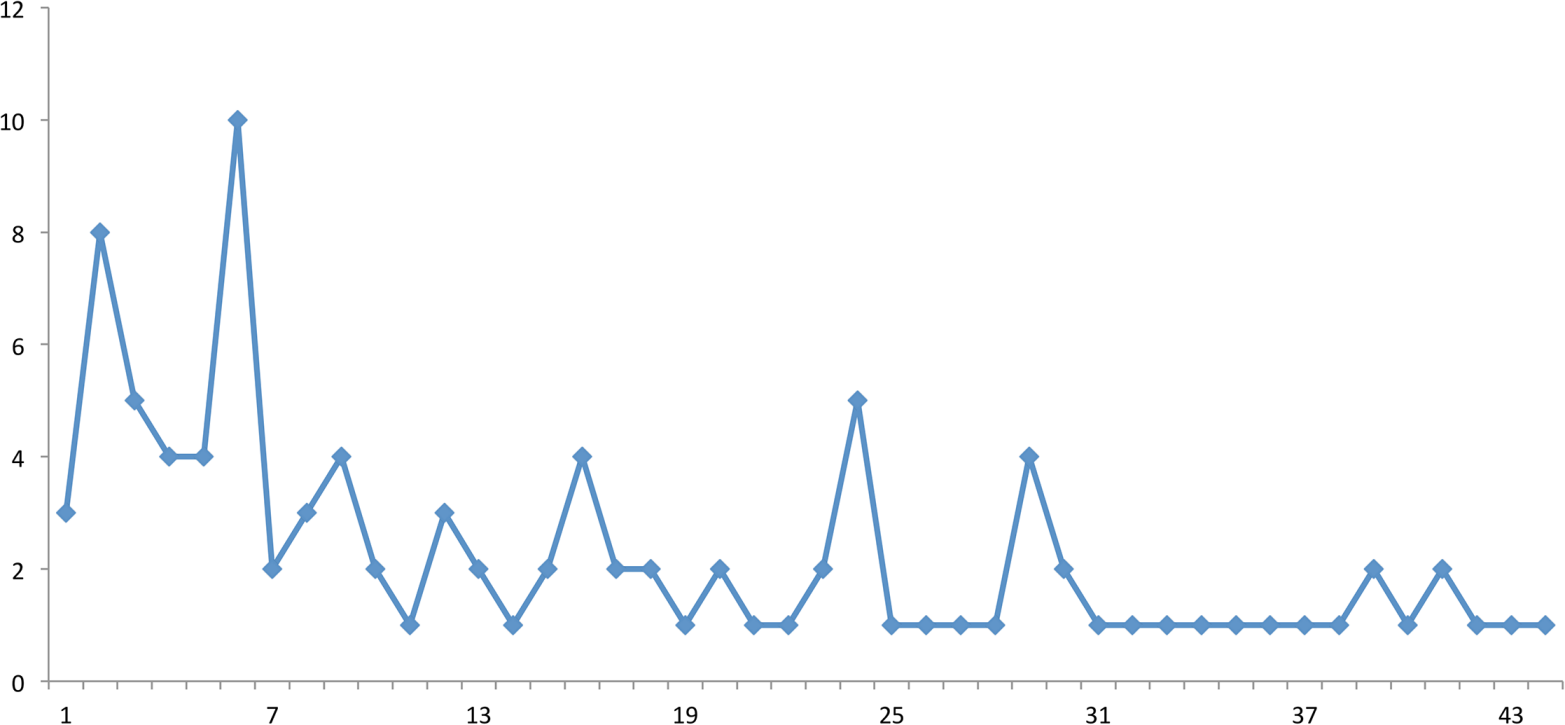
Number of open reviews (y-axis) per manuscript (consecutive manuscript number on x-axis).

Some 59 individual referees posted online reviews and the median (range) number of reviews posted per reviewer was 1 (1–15). The number of reviews via the online system declined over time (Fig 3). Thirty-eight of the 44 manuscript were rejected by the editor, while the authors of 6 (9%) manuscripts were invited to submit a revision of the paper.

### Review quality

The mean (SD) review quality scores are shown in Table 3. Agreement between the two individual assessors was low to medium, κ = 0.06–0.33. Mean scores were significantly lower in all aspects for online reviews than for conventional reviews. Summarised values for both assessors were 2.13 (± 0.75) for online reviews and 2.84 (± 0.71) for conventional reviews (P<0.001). The mean (SD) BJS score of the editors was 2.35 (± 0.74) for open reviews and 3.52 (±0.58) for conventional reviews (P<0.001).

**Table 3:** Summary of open and regular peer review scores. Means (SD).

	Regular peer review (n = 115)				Open peer review (n = 100)				
	Assessor A	Assessor B	Overall	κ	Assessor A	Assessor B	Overall	κ	P
**Importance**	2.99 (0.99)	2.25 (1.04)	2.62 (1.08)	0.12	2.13 (1.21)	2.07 (1.23)	2.10 (1.22)	0.26	0.001
**Originality**	2.96 (1.18)	2.43 (1.32)	2.69 (1.28)	0.25	1.79 (1.14)	1.98 (1.22)	1.88 (1.18)	0.30	<0.001
**Strengths/ weaknesses**	3.39 (0.92)	2.83 (1.12)	3.11 (1.06)	0.23	2.49 (1.23)	2.59 (1.19)	2.54 (1.21)	0.23	<0.001
**Presentation**	2.57 (0.90)	1.87 (1.16)	2.22 (1.09)	0.16	2.15 (1.10)	1.56 (0.99)	1.86 (1.09)	0.16	0.017
**Suggested changes**	3.21 (0.92)	3.10 (1.05)	3.15 (0.99)	0.33	2.40 (1.24)	2.62 (1.23)	2.51 (1.24)	0.33	<0.001
**Substantiation**	3.52 (0.76)	2.08 (1.19)	2.79 (1.22)	0.06	1.46 (0.92)	1.63 (0.95)	1.55 (0.94)	0.06	<0.001
**Interpretation**	3.27 (1.03)	1.97 (1.12)	2.62 (1.26)	0.11	1.74 (0.99)	1.31 (0.73)	1.52 (0.89)	0.11	<0.001
**Tone**	3.64 (0.69)	3.32 (0.65)	3.48 (0.69)	0.18	2.98 (0.89)	3.15 (0.72)	3.07 (0.81)	0.18	<0.001
**Sum**	3.19 (0.56)	2.48 (0.66)	2.84 (0.71)	-	2.15 (0.76)	2.12 (0.73)	2.13 (0.75)	-	<0.001
**Editors score**	-	-	3.52 (0.58)	-	-	-	2.35 (0.74)	-	<0.001

K agreement between assessor A and B. p, Student’s t-test.

### Post-trial questionnaire

The post-trial questionnaire (S3 Fig) was sent to all participating authors (n = 110). Only ten participants answered; of these, seven stated that they read the reviews via the experimental system, six thought them helpful and all ten would consider participating again.

## Discussion

In this study, feasibility, advantages and disadvantages of an experimental online forum peer review system in the setting of a high-quality general surgical journal were assessed. The pre-trial survey suggested that a majority of authors and reviewers were willing to participate in a study exploring this new method of peer review. The actual participation rate of authors in the study was 58%, with a median of one online review per manuscript. The quality of the online reviews varied considerably, but was significantly lower than conventional review. Given the large number of potential reviewers who were invited, the participation rate was very low.

In the pre-trial survey, previous submissions/publications, male gender and English as a first language were factors associated with willingness to participate. It is possible that those surveyed, who were all BJS reviewers, differed from authors in these aspects and that worries about the benefits of the online review process and satisfaction with the current system led authors to decline participation. It is also possible that some degree of suspicion around a new system precluded authors from taking part, and if this system became more common and/or accepted, participation rate would increase. Still, over half of the authors submitting during the trial period participated. In this regard, it must be noted that questions 9 and 10 of the survey were somewhat leading (S2 Fig), possibly giving the surveyed a negative view of open review, and that if this item had been rephrased, different responses might have been received.

Despite mailing an invitation to submit a review to more than 7000 referee account holders in the BJS ScholarOne system, we only received reviews from 59 individual reviewers. This has to be considered a disappointingly low rate. This could be due to the invitation itself: reviewers might be more attracted, and feel more responsible, if they receive a personal email from one of the editors than from an impersonal mass mail. Another reason could be a general difficulty in getting reviews. The intention was also to draw reviewers and readers into meaningful online debate about the work under review. The finding that the number of online reviewers declined with time also points towards a loss of interest of the potential reviewers and lack of resources used to win potential participants.

The lack of time and incentives for the reviewer have been pointed out previously[24] and other solutions such as remuneration, by economic or academic means, have been proposed. Cash, discounts for subscriptions or books with the publisher or continuous medical education (CME) points might increase the attraction to review [25].

Placing emphasis on reviewing experience when assigning academic posts within universities and medical schools might also increase the incentive to review. An interesting idea is to create a reviewer index [26, 27] which would be an objective, transparent way of highlighting the important work carried out by reviewers.

The low participation rate might also relate to a lack of competence in a specific field, real or perceived, on the part of the potential reviewer. Surgery and surgical science are, as all branches of medicine, growing more specialised every year. Most manuscripts submitted to BJS deal with a very specific area, in which only a fraction of the invited reviewers might be expected to have competence.

An open online system is attractive because of its transparency and potential to increase communication between reviewers and authors, but, in the present study, some potential reviewers might have refrained from reviewing because their names would have been made public. Thus, non-anonymity might be one reason for the low rate of open peer review. Even if anonymous, online peer review increases transparency, and there is no evidence that unmasking either authors’ or reviewers’ identities increases peer review quality [28].

Medicine has been slow to take on pre-print publication, which can be considered a special case of open peer review. In areas such as physics, statistics, engineering and mathematics, there are several online pre-print servers, on which authors display their work in progress and receive commentaries from the scientific community. For instance, arXiv.org, which started as early as the 1990s, currently houses over 1,000,000 openly available pre-prints. Many pre-print servers have moderators that function as editors, reviewing submissions, turning down those that are off-topic or simply not scientific papers. Some pre-print servers also make use of endorsers, who are authors in the field, who have the authority to allow unauthorised (non-endorsed) authors to submit papers, thus functioning as peer reviewers.

Recently, there have been some highly publicised, serious cases of fraudulent research. The content of individual papers, journals and publishing houses and review systems have been the subject of fraud and cheating. Getting papers published is, for many academics, directly related to career, status and income, and there is a risk that an online system could be misused. In this light, the authors’ perceived fear of having ideas stolen and plagiarised should be taken seriously. The post-trial survey was only answered by ten authors, which precludes any firm conclusions.

The quality of online review reports as judged using both a validated review quality tool and the conventional BJS scoring system was significantly lower than the quality of the conventional reviews; hence, one may conclude that online reviews cannot replace conventional, invited reviews.

Some limitations of the present study need to be mentioned. One is the lack of randomisation and a control group. The present results might not be applicable to all types of journals; for instance, if a similar study was performed on a strictly open access journal, it might yield different results. Further, the inter-rater variability between the raters using the validated review quality tool was higher than previously reported. Apart from the pre-trial survey, a more extensive engagement strategy could have been employed to increase author and reviewer participation. Such a strategy could include presentation at meetings and advertising in printed, online and social media. Once such a system is up and running and engagement is ensured, papers could come to the editor in a more mature and refined product which has been evaluated from multiple perspectives. Online peer review may not be focussed so much on the scientific credibility of a paper but may provide a better insight in the timeliness, attractive value, desirability and possible impact of a manuscript for the scientific community.

## Supporting information

S1 Fig Standard BJS referee sheets for peer review. (PDF)

S2 Fig Pre-trial questionnaire. (PDF)

S3 Fig Post-trial questionnaire. (PDF)

S1 Text RESEARCH_CHECKLIST_updated20170310. docx.
(DOCX)

S1 Data allpretrialdata encoded 20151105. dta.
(DTA)

S2 Data openreviewscores maandrv 20151108. dta.
(DTA)

S3 Data Peer review trial final data. xlsx.
(XLSX)

S4 Data regularscores maandrv 201501109. dta.
(DTA)
