# Supplementary material for: A prospective study on an innovative online forum for peer reviewing of surgical science
Source: PLoS One. 2017 Jun 29;12(6):e0179031. doi: 10.1371/journal.pone.0179031 (PMC5491000; doi:10.1371/journal.pone.0179031)
Supplement: S1 Fig — (PDF) [file pone.0179031.s001.pdf]

Title:	xxxxxxx
Manuscript ID:	xxxxxxx
Authors:	xxxxxxx
Wiley - Manuscript type:	xxxxxxx
Date Submitted:	xxxxxxx
Total Time in Review:	xxxxxxx

Status: EO: McFarlane, Kirsty

Under Review

HTML PDF Supplementary Files Abstract External Searches

Manuscript Structure (please answer either yes/no/not applicable)	Yes	No	N/A
**Abstract**			
Does the summary give an adequate picture of the paper overall?	○	○	○
**Introduction**			
Are the aims and objectives of the paper set out *clearly*?	○	○	○
Are the aims and objectives of the paper set out *succinctly*?	○	○	○
**Materials and Methods**			
Are these clearly explained?	○	○	○
Are the methods easily understood?	○	○	○
**Results**			
Is the cohort size appropriate?	○	○	○
Are the statistical methods appropriate?	○	○	○
Are statistics necessary?	○	○	○
**Discussion and Conclusions**			
Are the conclusions clearly stated?	○	○	○
Are the conclusions justified?	○	○	○
Are there any ethical issues to be addressed?	○	○	○
Are the conclusions adequately discussed against the background of current health issues?	○	○	○

Assessment (please grade between excellent and very poor)

Novelty/originality

Clinical importance

Scientific importance

Analysis of data

Presentation/grammar/style (including illustrations, tables etc.)

Manuscript Structure

Length of article is:

Number of tables:

Number of figures is:

req Is this article worthy of a Commentary?

If yes, would you be prepared to write it?

req **Recommendation**

- A - Excellent, must publish
- B - Good but needs minor correction
- C - Correctable deficits, probably publish
- D - Major faults, reject
- E - Very poor, reject outright

Comments

req Confidential Comments to the Editor

reqComments to the Author

Important to complete
