# Supplementary material for: A prospective study on an innovative online forum for peer reviewing of surgical science
Source: PLoS One. 2017 Jun 29;12(6):e0179031. doi: 10.1371/journal.pone.0179031 (PMC5491000; doi:10.1371/journal.pone.0179031)
Supplement: S2 Fig — (PDF) [file pone.0179031.s002.pdf]

BJS

BJS peer review survey

Welcome to the BJS peer review survey

Dear participant,

We would like to thank you for completing this survey, which addresses an important and crucial topic for all scientific journals: the review process.

BJS intends to perform a randomized trial addressing a potential improvement to the standard peer review process. In order to give you a better understanding, we briefly explain the novel approach in the following paragraph.

As usual, a submitted manuscript is preliminarily screened by an assigned editor and sent to three experts for standard peer review. The manuscript, including the referees' reports, are posted online on a secured website for a period of two weeks. The referees' comments will be anonymous unless permission is granted. Subscribers to BJS, including the authors and referees, will be able to review these documents for critical appraisal and are invited to give comments and pose questions. As usual, the authors are expected to respond to the referees' comments, but also to the comments and questions posted in the online website. After two weeks the editor will take a decision, as in the current peer review system.

BJS

BJS peer review survey

1. What is your age?

- ○ <30
- ○ 30-40
- ○ 40-50
- ○ 50-60
- ○ >60

BJS

BJS peer review survey

2. What is your sex?

- ○ Male
- ○ Female

BJS

BJS peer review survey

3. Is English your first language?

- ○ Yes
- ○ No

BJS

4. How many papers have you previously submitted and/or published?

- ◯ None
- ◯ 1-5
- ◯ 5-20
- ◯ >20

BJS

BJS peer review survey

5. Have you previously submitted and/or published a paper to/in the BJS?

- ◯ Yes
- ◯ No

BJS

BJS peer review survey

6. Do you review for biomedical journal(s)?

- ◯ No
- ◯ <10 times per year
- ◯ 10-20 times per year
- ◯ >20 times per year

BJS

BJS peer review survey

7. The BJS is considering an experiment in which submitted manuscripts will be randomized to standard peer review or to standard peer review with the addition of an online, open review, where readers may post their comments.

Would you submit your paper to BJS under these conditions?

- ◯ Yes
- ◯ No
- ◯ Unsure

BJS

BJS peer review survey

8. If not, please list your reason(s) for not participating.

BJS

BJS peer review survey

9. A concern with open peer review is that authors may not be keen to share their ideas before the paper is accepted. Would this have an effect upon your decision to submit a manuscript to BJS?

- More likely to submit
- Less likely to submit
- No difference

BJS

BJS peer review survey

10. Others might feel that open peer review devalues their work. Would this have an effect upon your decision to submit a manuscript to BJS?

- More likely to submit
- Less likely to submit
- No difference

BJS

BJS peer review survey

11. If the BJS put up papers for online review, would you take the opportunity to comment on them?

- ◯ Yes
- ◯ No
- ◯ Unsure
