# Supplementary material for: A prospective study on an innovative online forum for peer reviewing of surgical science
Source: PLoS One. 2017 Jun 29;12(6):e0179031. doi: 10.1371/journal.pone.0179031 (PMC5491000; doi:10.1371/journal.pone.0179031)
Supplement: S3 Fig — (PDF) [file pone.0179031.s003.pdf]

BJS Peer Review Trial Post-Trial Survey

1. What was your main reason for participating?

2. How well did the submission process work?

- Very poorly
- Poorly
- Adequately
- Well
- Very well

3. How easy was it to understand the description of the open peer review trial?

- Very difficult
- Difficult
- Acceptable
- Easy
- Very easy

4. Did you read the open peer reviews?

- Yes
- No
- Not applicable

5. If so, how helpful were they to you when revising your manuscript?

- Very unhelpful
- Unhelpful
- Helpful
- Very helpful
- Not applicable

6. Were you negatively impacted due to participating in the open peer review?

- Yes
- No

If yes, please specify

7. Would you consider participating again?

- No
- Maybe
- Yes

Thank you very much for your time and for your support of the journal.

The BJS Editorial Team
