# Supplementary material for: A prospective study on an innovative online forum for peer reviewing of surgical science
Source: PLoS One. 2017 Jun 29;12(6):e0179031. doi: 10.1371/journal.pone.0179031 (PMC5491000; doi:10.1371/journal.pone.0179031)
Supplement: S1 Text — docx. (DOCX) [file pone.0179031.s004.docx]

# RESEARCH CHECKLIST for “A prospective study on an innovative online forum for peer reviewing of surgical science”

Martin Almquist, Regula S von Allmen, Dan Carradice, Steven J. Oosterling, Kirsty McFarlane, Bas Wijnhoven

1. Study protocol. The complete study protocol is found below.
2. This trial did not involve any patients, and is probably best described as an observational study. Therefore, the STROBE guidelines were followed when designing and reporting the study. The manuscript has been checked against the STROBE checklist and all items are covered. The STROBE checklist is included in this research checklist, please see below.

## STUDY PROTOCOL

# Peer Review Trial

**STUDY PROTOCOL**

Last update: 07-02-2015

Martin Almquist, Regula S. von Allmen, Dan Carradice, Steven Oosterling, Bas P.L. Wijnhoven

## Background

Peer review is important for authors, editors and the whole scientific community. A survey of over 4000 international authors revealed that whilst 91% believe that peer review has improved their work, 2/3 feel that conventional peer review can be improved.^1, 2^ Current peer review models can induce bias ^3-11^, fail to identify error and fraud ^12-15^ and suppress innovation.^16^ Thus, new and innovative models of peer review are needed. Therefore, we propose an open peer review system where a wider audience is invited to review papers online. Due to uncertainty about the acceptance of such a novel peer review process, a feasibility study will be performed at first, where authors are invited consecutively to subject their submitted papers to online open review in addition to standard peer review.

## Aim

- To explore whether authors, editors and reviewers are prepared to participate in a peer review process via an online open peer review system, in order to prepare for a larger randomised controlled trial
- To explore the acceptability of this process to authors, reviewers and editors
- To evaluate the advantages, disadvantages and practical issues associated with such a process

## Hypotheses

**Null hypothesis**

- The open peer review process is as feasible in terms of reviewers’ and editors’ engagement and results in referee reports of equally good quality.

**Alternative hypothesis**

- The open peer review is not feasible, because participation rates are too low and/or there is too little perceived benefit by authors, reviewers and/or editors

## Methods/Design

**Setting**

This study will be performed by the assistant editors of BJS. BJS will act as sponsor of the trial.

**Study design**

An observational feasibility trial. Included papers will undergo conventional peer review and simultaneous open peer review.

**Eligibility assessment and consent**

Submitted manuscripts are subject to internal reviewing. All manuscripts that are not immediately rejected will be eligible for enrolment. Informed consent is asked for at the time of submission of the paper online via Scholar One. Inclusion into the study is only performed once an informed consent by the corresponding author is given using an electronic signature.

**Standard peer review process**

In the standard process, the manuscripts are subject to external reviewing only. The handling editor sends the paper to two to four referees of his/her choice. The referees are asked to return their reports to the editorial office within two weeks. Referees use the current online evaluation forms. After submission of at least two referee reports, the handling editor scores the reports according to standard BJS policy and according to the existing specific review quality instrument as described below.

Eventually, the handling editor makes a decision: accept paper without changes needed, invitation to resubmit the paper following major or minor revisions or rejection of the manuscript. This review process takes on average 22 days. By default, the open peer review is single-blinded in which the reviewer’s name is not disclosed to the authors.

**Open peer review through online system**

Following submission a manuscript will simultaneously go through standard peer review and also to the innovative process under study.

At the same time, the manuscript will be posted on a dedicated website for open access reviewing using Google Docs.

A mail is sent out every fortnight inviting potential referees as outlined below to review posted papers Abstracts of posted papers are included in this mail as well as a direct link to the paper and the online website. The email also contains an unsubscribe link.

Referees who like to review the paper on the open website are asked to adhere to the same online evaluation forms (standard referee sheet) as in the standard peer review process. It is also mentioned that the handling editor makes the final decision and that the reviewers should not mention in their reports whether the manuscript merits publication or not.

Once the review has been submitted, it will be immediately posted on the website and will be accessible to authors and other invited reviewers, i.e. open. By default, reviewers’ names and affiliations will be shown; anonymous reviewing is not allowed. Authors are notified via e-mail when a new review is posted.

After a period of 21 days, the manuscript and corresponding referee reports will be removed from the website and stored at manuscript central?).

**Final decision**

The responsible editor handles the manuscript in a standard way; he/she makes a decision, based on the standard peer review blinded to the comments from the open peer review process. Once the final decision is made, he/she then evaluates the online open referee reports according to standard BJS policy. The referee reports from the open review process are available to the author and can be used to improve the paper when resubmission is requested (first revision, i.e. R1) or for submission to another journal.

The assistant editors however, score all referee reports (invited reports and those from the open review process) according to the review quality instrument as described below. The assessors are not blinded to the source of the referee reports (invited *versus* open).

**Copyright**

In order to secure the authors’ copyright during open peer review process, a line needs to be added at the end of every manuscript: “Copyright of the authors”. Once a paper is accepted, standard BJS policy will be applied and the corresponding author for the paper will receive an email prompting them to log in to Author Services where, via the Wiley Author Licensing Service (WALS), they will be able to complete the license agreement on behalf of all authors on the paper.

**Invitation of potential referees**

BJS subscribers and all physicians that are affiliated to BJS by their organisations (e.g. by membership number) as well as existing BJS referees are invited

- [The Vascular Society of Great Britain and Ireland](http://www.vascularsociety.org.uk/)
- [Association of Surgeons of Great Britain and Ireland](http://www.asgbi.org.uk/)
- [Society of Academic and Research Surgery](http://surgicalresearch.org.uk/)
- [Swedish Surgical Society](http://www.svenskkirurgi.se/skf/v2/)
- [Swiss Society of Surgery](http://sgc-ssc.ch/)

A newsletter (e-mail) is sent to the identified potential referees alerting them about the papers that are on the online website with details about the main surgical field (upper and lower gastrointestinal surgery, pancreatic, liver surgery, breast surgery, vascular, endocrine surgery, trauma surgery).

**Review quality instrument version 4**

An established validated review quality instrument consisting of seven items formulated as questions that relate to importance of the research question, originality, method, presentation, constructiveness of the comments, substantiation of comments and interpretation of results is used. Each of the seven items scores in a five-point Likert scale from 1 (=poor) to 5 (=excellent). An additional eighth question assessed the overall tone of the review ranging from abusive to courteous. A total score is based on the mean of the eight items scores.17 (Figure 1)

**Outcome assessment**

- **Main outcome**
  - Quality of referee reports (based on the used review quality instrument)
- **Secondary outcomes:**
  - Participation rate, i.e. number of reviews and/or author comments per paper in open peer review
  - **Quality of referee reports (based on the BJS scoring system for peer review quality)**
  - **Reviewer’s recommendation regarding publication (**accept paper without changes needed, publish with major revision, publish with minor revision, reject.)

**Study time frame**

Consecutive papers are included within a predefined timeframe.

- - Start date: 1^st^ April 2015
  - End date: 31^st^ May 2015

This will yield approximately 170 papers (taking into account that roughly 150 papers are submitted to BJS each month, that roughly n=25 papers per month are rejected outright without undergoing formal peer review and that roughly 70% of authors will provide informed consent for the study).

**Costs**

By using Google Docs, which is free of charge only minimal costs will be incurred. If any unforeseen costs are encountered, a timely notification should be sent to Mr. Jonothan Earnshaw, chief editor of BJS.

**Data management and analysis**

The quality of the referee reports between the two different review processes (standard *versus* open) will be compared using non-parametric statistics.

Number of open reviews per paper and number of comments per paper will be calculated.

## Timeline

- Discussion with BJS digital manager regarding website.
- Approval of protocol at BJS meeting in March 2015 (Birmingham) – done!
- Testing of the website during March: a pilot study with 10 papers will be necessary, for which the open peer review process will be simulated by the assistant editors only without involving external referees.
- Timely start of the trial April 1^st^ 2015
- Presentation of preliminary results at BJS meeting in May 2015 (Maastricht)
- Report of study results at BJS meeting in July (London).
- Publication is expected to be finalised in autumn 2015.

## Working group

Martin Almquist

Regula von Allmen

Dan Carradice

Steven Oosterling

Bas Wijnhoven,

Cherrie Alison with support from BJS

**References**

1. Sense about Science. Peer Review Survey. <http://wwwsenseaboutscienceorg/data/files/Peer_Review/Peer_Review_Survey_Final_3pdf> [serial on the Internet]. 2009.

2. Mulligan A, Hall L, Raphael E. Peer review in a changing world: An international study measuring the attitudes of researchers. J Am Soc Inf Sci. 2013;64(1):132-61.

3. Ceci SJ, Peters DP. Peer review--a study of reliability. Change. 1982 Sep;14(6):44-8.

4. Ceci SJ, Williams WM. Understanding current causes of women's underrepresentation in science. Proceedings of the National Academy of Sciences of the United States of America. 2011 Feb 22;108(8):3157-62.

5. Emerson GB, Warme WJ, Wolf FM, Heckman JD, Brand RA, Leopold SS. Testing for the presence of positive-outcome bias in peer review: a randomized controlled trial. Archives of internal medicine. 2010 Nov 22;170(21):1934-9.

6. Lee KP, Boyd EA, Holroyd-Leduc JM, Bacchetti P, Bero LA. Predictors of publication: characteristics of submitted manuscripts associated with acceptance at major biomedical journals. The Medical journal of Australia. 2006 Jun 19;184(12):621-6.

7. Link AM. US and non-US submissions: an analysis of reviewer bias. Jama. 1998 Jul 15;280(3):246-7.

8. Lynch JR, Cunningham MR, Warme WJ, Schaad DC, Wolf FM, Leopold SS. Commercially funded and United States-based research is more likely to be published; good-quality studies with negative outcomes are not. The Journal of bone and joint surgery American volume. 2007 May;89(5):1010-8.

9. Olson CM, Rennie D, Cook D, Dickersin K, Flanagin A, Hogan JW, et al. Publication bias in editorial decision making. Jama. 2002 Jun 5;287(21):2825-8.

10. Opthof T, Coronel R, Janse MJ. The significance of the peer review process against the background of bias: priority ratings of reviewers and editors and the prediction of citation, the role of geographical bias. Cardiovascular research. 2002 Dec;56(3):339-46.

11. Wenneras C, Wold A. Nepotism and sexism in peer-review. Nature. 1997 May 22;387(6631):341-3.

12. BBC. Profile: Hwang Woo-suk. <http://newsbbccouk/1/hi/world/asia-pacific/4554704stm> [serial on the Internet]. 2009.

13. Bohannon J. Who's afraid of peer review? Science. 2013 Oct 4;342(6154):60-5.

14. Godlee F, Gale CR, Martyn CN. Effect on the quality of peer review of blinding reviewers and asking them to sign their reports: a randomized controlled trial. Jama. 1998 Jul 15;280(3):237-40.

15. Wakefield AJ, Murch SH, Anthony A, Linnell J, Casson DM, Malik M, et al. Ileal-lymphoid-nodular hyperplasia, non-specific colitis, and pervasive developmental disorder in children. Lancet. 1998 Feb 28;351(9103):637-41.

16. Campanario JM. Commentary: On Influential Books and Journal Articles Initially Rejected Because of Negative Referees' Evaluations. Science Communication. 1995;16(3):304-25.

17. van Rooyen S, Godlee F, Evans S, Black N, Smith R. Effect of open peer review on quality of reviews and on reviewers' recommendations: a randomised trial. Bmj. 1999 Jan 2;318(7175):23-7.

Figure 1:


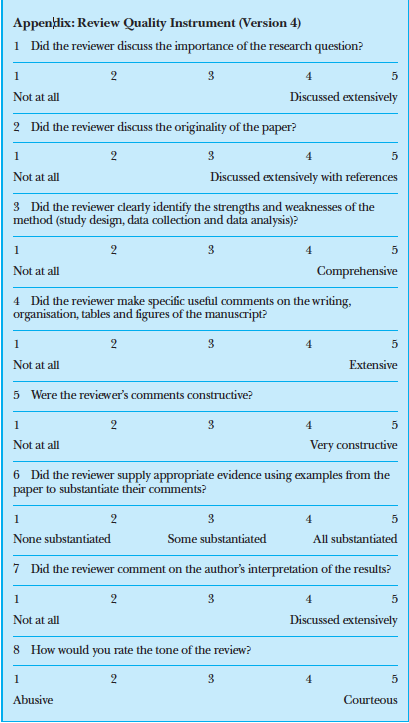


STROBE CHECKLIST STROBE Statement—checklist of items that should be included in reports of observational studies

|  | Item No | Recommendation |
| --- | --- | --- |
| **Title and abstract** | 1 | (*a*) Indicate the study’s design with a commonly used term in the title or the abstract TITLE PAGE/ABSTRACT PAGE 1/2 |
|  |  | (*b*) Provide in the abstract an informative and balanced summary of what was done and what was found ABSTRACT PAGE 2 |
| Introduction | | |
| Background/rationale | 2 | Explain the scientific background and rationale for the investigation being reported INTRODUCTION PAGE 5 |
| Objectives | 3 | State specific objectives, including any prespecified hypotheses INTRODUCTION PAGE 5 |
| Methods | | |
| Study design | 4 | Present key elements of study design early in the paper  METHODS PAGE 7-9 |
| Setting | 5 | Describe the setting, locations, and relevant dates, including periods of recruitment, exposure, follow-up, and data collection  METHODS PAGE 6-9 |
| Participants | 6 | (*a*) *Cohort study*—Give the eligibility criteria, and the sources and methods of selection of participants. Describe methods of follow-up  *Case-control study*—Give the eligibility criteria, and the sources and methods of case ascertainment and control selection. Give the rationale for the choice of cases and controls  *Cross-sectional study*—Give the eligibility criteria, and the sources and methods of selection of participants PAGE 7-9 |
|  |  | (*b*) *Cohort study*—For matched studies, give matching criteria and number of exposed and unexposed  *Case-control study*—For matched studies, give matching criteria and the number of controls per case |
| Variables | 7 | Clearly define all outcomes, exposures, predictors, potential confounders, and effect modifiers. Give diagnostic criteria, if applicable  PAGE 7-9 |
| Data sources/ measurement | 8* | For each variable of interest, give sources of data and details of methods of assessment (measurement). Describe comparability of assessment methods if there is more than one group  PAGE 7-9 |
| Bias | 9 | Describe any efforts to address potential sources of bias  PAGE 7-9 |
| Study size | 10 | Explain how the study size was arrived at  PAGE 7-9 |
| Quantitative variables | 11 | Explain how quantitative variables were handled in the analyses. If applicable, describe which groupings were chosen and why  PAGE 7-9 |
| Statistical methods | 12 | (*a*) Describe all statistical methods, including those used to control for confounding |
|  |  | (*b*) Describe any methods used to examine subgroups and interactions |
|  |  | (*c*) Explain how missing data were addressed |
|  |  | (*d*) *Cohort study*—If applicable, explain how loss to follow-up was addressed  *Case-control study*—If applicable, explain how matching of cases and controls was addressed  *Cross-sectional study*—If applicable, describe analytical methods taking account of sampling strategy |
|  |  | (*e*) Describe any sensitivity analyses  PAGE 7-9 |

Continued on next page

| Results | | |
| --- | --- | --- |
| Participants | 13* | (a) Report numbers of individuals at each stage of study—eg numbers potentially eligible, examined for eligibility, confirmed eligible, included in the study, completing follow-up, and analysed PAGE 10-12, FIGURE 2 |
|  |  | (b) Give reasons for non-participation at each stage  PAGE 10-12, FIGURE 2 |
|  |  | (c) Consider use of a flow diagram  FIGURE 2 |
| Descriptive data | 14* | (a) Give characteristics of study participants (eg demographic, clinical, social) and information on exposures and potential confounders PAGE 10-12 |
|  |  | (b) Indicate number of participants with missing data for each variable of interest PAGE 10-12, TABLE 1 |
|  |  | (c) *Cohort study*—Summarise follow-up time (eg, average and total amount) |
| Outcome data | 15* | *Cohort study*—Report numbers of outcome events or summary measures over time |
|  |  | *Case-control study—*Report numbers in each exposure category, or summary measures of exposure |
|  |  | *Cross-sectional study—*Report numbers of outcome events or summary measures PAGE 10-12, TABLE 1-3 |
| Main results | 16 | (*a*) Give unadjusted estimates and, if applicable, confounder-adjusted estimates and their precision (eg, 95% confidence interval). Make clear which confounders were adjusted for and why they were included RESULTS PAGE 10-12, TABLE 1-3 |
|  |  | (*b*) Report category boundaries when continuous variables were categorized RESULTS PAGE 10-12, TABLE 1-3 |
|  |  | (*c*) If relevant, consider translating estimates of relative risk into absolute risk for a meaningful time period |
| Other analyses | 17 | Report other analyses done—eg analyses of subgroups and interactions, and sensitivity analyses |
| Discussion | | |
| Key results | 18 | Summarise key results with reference to study objectives DISCUSSION PAGE 13 |
| Limitations | 19 | Discuss limitations of the study, taking into account sources of potential bias or imprecision. Discuss both direction and magnitude of any potential bias DISCUSSION PAGE 15 |
| Interpretation | 20 | Give a cautious overall interpretation of results considering objectives, limitations, multiplicity of analyses, results from similar studies, and other relevant evidence DISCUSSION PAGE 15 |
| Generalisability | 21 | Discuss the generalisability (external validity) of the study results DISCUSSION PAGE 14-15 |
| Other information | | |
| Funding | 22 | Give the source of funding and the role of the funders for the present study and, if applicable, for the original study on which the present article is based ACKNOWLEDGEMENTS PAGE 15-16 |

*Give information separately for cases and controls in case-control studies and, if applicable, for exposed and unexposed groups in cohort and cross-sectional studies.

**Note:** An Explanation and Elaboration article discusses each checklist item and gives methodological background and published examples of transparent reporting. The STROBE checklist is best used in conjunction with this article (freely available on the Web sites of PLoS Medicine at http://www.plosmedicine.org/, Annals of Internal Medicine at http://www.annals.org/, and Epidemiology at http://www.epidem.com/). Information on the STROBE Initiative is available at www.strobe-statement.org.
